# Divergent gene expression in the conserved dauer stage of the nematodes *Pristionchus pacificus* and *Caenorhabditis elegans*

**DOI:** 10.1186/1471-2164-13-254

**Published:** 2012-06-19

**Authors:** Amit Sinha, Ralf J Sommer, Christoph Dieterich

**Affiliations:** 1 Max-Planck Institute for Developmental Biology, Department for Evolutionary Biology, Spemannstrasse 37, 72076, Tübingen, Germany; 2 Berlin Institute for Medical Systems Biology (BIMSB) at the Max Delbrück Center for Molecular Medicine Berlin, Robert-Röβle-Str. 10, 13125, Berlin, Germany

**Keywords:** Dauer larvae, Developmental systems drift, Transcriptomics, Evolution of gene regulation, Horizontal gene transfer

## Abstract

**Background:**

An organism can respond to changing environmental conditions by adjusting gene regulation and by forming alternative phenotypes. In nematodes, these mechanisms are coupled because many species will form dauer larvae, a stress-resistant and non-aging developmental stage, when exposed to unfavorable environmental conditions, and execute gene expression programs that have been selected for the survival of the animal in the wild. These dauer larvae represent an environmentally induced, homologous developmental stage across many nematode species, sharing conserved morphological and physiological properties. Hence it can be expected that some core components of the associated transcriptional program would be conserved across species, while others might diverge over the course of evolution. However, transcriptional and metabolic analysis of dauer development has been largely restricted to *Caenorhabditis elegans*. Here, we use a transcriptomic approach to compare the dauer stage in the evolutionary model system *Pristionchus pacificus* with the dauer stage in *C. elegans*.

**Results:**

We have employed Agilent microarrays, which represent 20,446 *P. pacificus* and 20,143 *C. elegans* genes to show an unexpected divergence in the expression profiles of these two nematodes in dauer and dauer exit samples. *P. pacificus* and *C. elegans* differ in the dynamics and function of genes that are differentially expressed. We find that only a small number of orthologous gene pairs show similar expression pattern in the dauers of the two species, while the non-orthologous fraction of genes is a major contributor to the active transcriptome in dauers. Interestingly, many of the genes acquired by horizontal gene transfer and orphan genes in *P. pacificus*, are differentially expressed suggesting that these genes are of evolutionary and functional importance.

**Conclusion:**

Our data set provides a catalog for future functional investigations and indicates novel insight into evolutionary mechanisms. We discuss the limited conservation of core developmental and transcriptional programs as a common aspect of animal evolution.

## Background

The primary mechanisms of an organism to respond to changing environmental conditions are alterations of gene expression profiles and the formation of dormant or dormant-like developmental stages. Nematodes are found in great numbers and species richness in most ecosystems on earth. This omnipresence is attributed to the evolution of a potent life history strategy to respond to changing environments [1]. Under favorable conditions nematodes such as *Caenorhabditis elegans* and *Pristionchus pacificus* undergo direct development, which can be completed within 3–4 days. In contrast, under harsh environmental conditions such as food scarcity, high temperature and high population density, these nematodes form an arrested developmental stage, the so-called dauer larvae [2].

Dauer larvae are stress-resistant and “non-aging” and they facilitate the survival and dispersion of the organism [3]. Entry into the dauer stage represents the major life history response of nematodes to escape unfavorable environmental conditions [1]. This developmental switch is accompanied with remarkable transcriptional and metabolic changes as shown in several studies in *C. elegans*[4-8]. Since the dauer larva is an ecologically induced, conserved developmental stage found in many species of free-living nematodes, it can be expected that some genetic components involved in development might be conserved, while those facilitating ecological adaptation might diverge across species.

However, the similarity and divergence patterns of nematode transcriptomes have never been systematically investigated in the context of dauer larvae. While several transcriptional studies in *C. elegans* provide a platform for comparative studies [5,6,8-14], little is known in other free-living nematodes. Here we present a comparative transcriptomic approach to characterize the expression profiles of the dauer and dauer exit stages in *C. elegans* and *P. pacificus.* We measured transcriptome-wide expression changes with the help of the Agilent microarray platform, and use this data to identify the extent and nature of similarity or differences in the dauer-associated transcriptomes in the two nematode species.

*C. elegans* was the first metazoan to have its complete genome sequenced [15]. Ever since, *C. elegans* has been at the forefront of embracing novel “omics” technologies, including transcriptomics [16,17], proteomics [18,19] and RNAseq [20]. Comparative genomics is a powerful tool to address elementary questions in evolutionary developmental biology, such as what components of the regulatory "tool-kit" are conserved [21]. By now, the genome of six additional nematodes and many more transcriptomic studies have been reported (for review see [22]). Comparative transcriptomics opens up new vistas on how regulatory "tool-kit" components are employed to generate different morphologies across species [21,23]. In nematodes, comparative transcriptomic approaches provide a powerful way to analyze whole body responses to changes in the environment or the exposure to pathogens. The dauer stage represents a whole body response of the nematode to changing environmental conditions and is ideally suited for comparative transcriptomics studies.

We compare *C. elegans* to *P. pacificus*, an established model for comparative developmental biology, evolutionary biology and ecology [24]. Forward and reverse genetic as well as transgenic techniques have been established in *P. pacificus,* its genome has been sequenced [25] and its proteome has been analyzed by tandem mass spectrometry [26]. The sequencing of the *P. pacificus* genome revealed many important and unexpected features, such as a substantially larger size and a higher number of predicted protein-coding genes when compared to *C. elegans*, the presence of horizontal gene transfer and the duplication of genes encoding enzymes involved in the detoxification of xenobiotics [25]. Many of these features have been discussed in the context of a specific association with scarab beetles, in which *P. pacificus* is found in the wild [27]. Specifically, *P. pacificus* exists exclusively as dauer larvae on the living beetle and only reproduces after the beetle´s death by feeding on microbes that develop on the carcass [27-29]. This specific, so-called necromenic lifestyle shows the unique importance of the dauer stage and asks for a detailed functional investigation of the associated transcriptional programs.

Two developmental processes that have been studied in great detail at the genetic and molecular level in *P. pacificus* are the formation of the vulva, the egg-laying structure of nematode females and hermaphrodites [30-32] and the regulation of the dauer development [33,34]. Surprisingly, the regulation of vulva development in *C. elegans* and *P. pacificus* employs different signaling pathways to control the formation of this homologous morphological structure. In dauer formation, the two transcriptional regulators DAF-12, a nuclear hormone receptor, and DAF-16, a forkhead transcription factor, are well conserved between *C. elegans* and *P. pacificus*[33,34]. *In P. pacificus,* upstream factors consisting of insulin and TGF-beta signaling pathways in *C. elegans* await future analysis. Similarly, it is unclear whether downstream targets that are activated by DAF-12 and DAF-16 are also evolutionarily conserved. To address this question, we have taken a transcriptomic approach to directly compare expression profiles of the dauer stage (0 h) and dauer-exit stage (12 h post recovery-induction) in *C. elegans* and *P. pacificus*.

Here we show that *P. pacificus* has a more dynamic transcriptome during the dauer to dauer-exit transition as compared to that in *C. elegans*. The expression profiles from the two species look surprisingly different with limited overlap and weak correlation between orthologous genes that are differentially expressed during the dauer stage and the dauer-recovery process. Within the conserved genes, functionally different GO classes and protein domains are enriched in each profile, most striking differences being observed in regulation of metabolism related genes. These results highlight the importance of transcriptomic studies in revealing functional divergence in downstream effectors of homologous developmental processes, despite conservation of upstream regulatory factors.

## Results

### Significant drop in RNA abundance in dauer larvae of *C. Elegans* and *P. Pacificus*

The dormant dauer stage in *C. elegans* is associated with a global repression of Pol-II based transcription, down to 11 - 17% of other stages [4]. We quantified the amount of total RNA per worm in the dauer and mix-stage samples and observed that dauers of *C. elegans* and *P. pacificus* contain approximately 20-fold less total RNA per worm as compared to the respective mix-stage sample. On average, ~100,000 dauer larvae yielded the same amount of total RNA as ~5,000 mixed stage worms. On top of this global transcriptional repression, we detected less mRNA in the total RNA of dauer larvae. The mRNA proportion is reduced to about half of the mix-stage levels in both species as measured by *in vitro* transcription (Additional file 1: Figure S1a). This global repression is expected to result in most of the genes being down regulated in a dauer versus mix-stage comparison. Our normalization strategy is based on differential weighing of spiked-in RNA probes (see Methods, Additional file 1: Figure S1b) and confirms this trend for both the species (Additional file 1: Figure S1c).

### The *P. Pacificus* transcriptome is more dynamic in the dauer to dauer-exit transition

The Agilent microarray technology enabled simultaneous expression profiling of 20,143 genes in *C. elegans* and 20,446 genes in *P. pacificus*. Our experimental setup contrasted gene expression in the dauer stage (time point 0 hour) with the dauer-exit samples (time point 12 hour) via a common reference sample (mixed stage) for each species. This so called “common reference” experimental design for microarray makes use of a species-specific pool of RNA as a common technical reference, and was chosen as it potentially facilitates an extension of this study to other time-points and conditions, if needed (see [35], Methods and Additional file 1: Figure S2 for details). We have chosen the 12-hour post induction time-point to enable comparisons with published expression profiling experiments [5]. We have chosen the same 12-hour time-point for the *P. pacificus* dauer exit samples after verifying that the post-dauer development follows similar kinetics in both species. We base this conclusion on the following morphological and developmental observations: First, most of the population (~90%) of the recovering worms from *C. elegans* as well as *P. pacificus* resume pharyngeal pumping within 3 hours after inducing dauer exit. Second, at the 12-hour time-point (the stage when we collect the dauer-exit samples), no discernible morphological differences are found between the recovered dauers from both species. Third, the recovering animals from both species enter a lethargus stage between 13 to 14 hours post-recovery [3]. Fourth, the worms that are allowed to develop further at 20^0^ C reach the next moult (L4 for *C. elegans,* J4 for *P. pacificus*) between 22 to 23 hours after induction of dauer exit. Finally, recovered worms from both species started laying eggs between 42 to 45 hours post-induction. Based on all these criteria, we consider the 12-hour post recovery induction time-point to be developmentally equivalent and comparable across the two species.

The number of genes, which are differentially expressed in the dauer to dauer exit transition (Table 1), is much larger in *P. pacificus* (4942 genes, Additional file 2: Table S1) than that seen in *C. elegans* (917 genes, Additional file 3: Table S2), suggesting more dynamic transcriptional changes accompanying dauer recovery in *P. pacificus*. Also, the number of up-regulated versus down-regulated genes in the dauers to dauer-exit comparison is much larger in *P. pacificus* than that in *C. elegans*, where the number of up- and down- regulated genes is essentially the same (Table 1). Thus *P. pacificus* dauers potentially require the activity of more genes to survive in their ecological niche than the dauer recovery stage.

**Table 1:** Differentially expressed genes in the dauer versus dauer-exit comparison

***P. pacificus*transcriptome is relatively more dynamic in dauer to dauer-exit transition (FDR corrected p-value < = 0.05)**				
	Up	Down	Total	Total genes on array
C. elegans	476	441	917	20143
P. pacificus	3545	1394	4939	20446

The Agilent arrays interrogate 20,446 genes for *P. pacificus* and 20,143 genes for *C. elegans*. We measure expression changes in the dauer to dauer-exit (12-hour time-point) transition using species-specific mix-stage sample as a common technical reference. Genes were called significantly differentially expressed (up- or down- regulated) based on a FDR corrected p-value cut-off of 0.05. *P. pacificus* transcriptome appears to be more dynamic during this transition.

### Signatures of conservation and divergence in the transcriptomes of the two species

**Figure 1:**
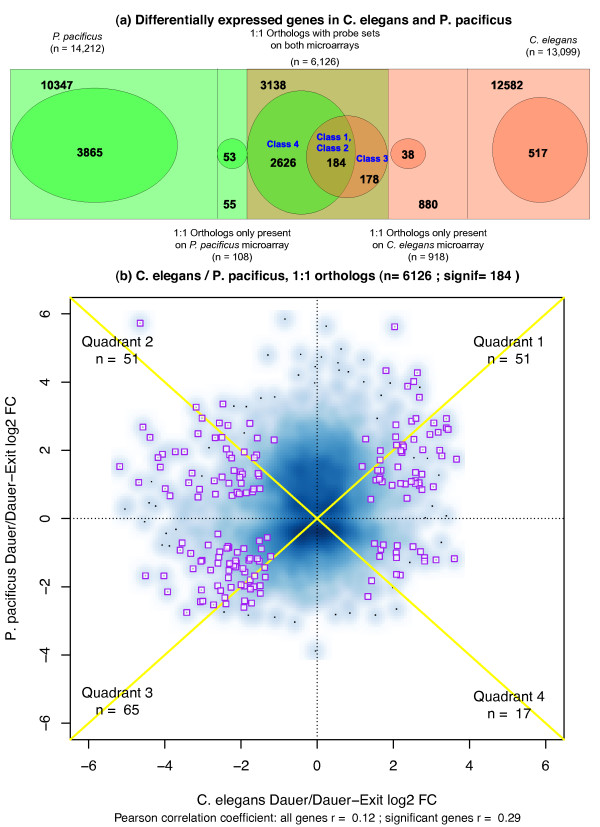
**Limited conservation in the dauer related transcriptomes of the two species.** (**a**) Overlap between genes differentially expressed in *P. pacificus* (green) and *C. elegans* dauers versus dauer-exit (12 hours) samples. The rectangular boxes represent the entire transcriptome on the array and their area of overlap represents the 1:1 orthologs between the two species, which are represented on both microarrays. The ovals represent the set of differentially expressed genes in each species. 184 orthologous gene pairs are called significantly differentially expressed in both the species, indicating limited conservation (Fisher's exact test p-value = 0.029). A substantial number of non-orthologous, species-specific genes are also differentially expressed in both the species. These gene sets are further analyzed by dividing them into distinct classes which are indicated in blue text (see main text for details) (**b**) Comparison of log2 expression fold changes for the set of 6,126 1:1 orthologs for the dauer versus dauer-exit comparison. Pearson's correlation coefficient over the entire set of 6,126 orthologs is r = 0.12, and increases to r = 0.29 for the 184 orthologs, which are significantly differentially expressed in both species (FDR corrected p-value < =0 0.05; purple boxes mark the significant orthologs, number per quadrant I - IV: 51 + 51 + 65 + 17 = 184).

Using pairwise best BLAST mapping, we identified 6,126 1:1 orthologous gene pairs in the two species, which are represented on both the arrays, while the remaining 14,212 *P. pacificus* genes and 13,099 *C. elegans* genes represent unresolved homology relations and lineage-specific genes. Within the 6,126 orthologs, we find that only 184 gene pairs are expressed at significantly different levels in the dauer versus dauer-exit comparisons of both species (FDR corrected p-value < 0.05) (Figure 1a). This small overlap is nonetheless statistically significant (Fisher’s exact test p-value = 0.029) indicating some “conservation” between dauer related genes in the two species.

The Pearson's correlation coefficient (r) between the fold changes of the 1:1 orthologs is r = 0.12 when calculated over all 6,126 orthologs, and increases to r = 0.29 when calculated on the 184 genes that are significantly differential in both species (Figure 1b). To further characterize the cross-species similarities and differences in transcriptomes, we group the set of 1:1 orthologs into the following classes:

(1) Class 1 = common orthologs that show a concordant pattern of differential regulation between the two species (up-regulated in both species, n = 51, quadrant 1, and down-regulated in both species n = 65, quadrant 3, Figure 1b. Total = 116 out of 184 common orthologs). These are expected to be the most conserved part of the transcriptomes across species (r = 0.88)

(2) Class 2 = orthologous genes that show a negative correlation in their direction of fold-change (n = 51, quadrant 2, and n = 17, quadrant 4, Figure 1b. Total = 68 out of total 184 common orthologs). This class of genes exhibits the most divergent expression pattern in the two transcriptomes (r = −0.80) and is comparable in size to class 1.

(3) Class 3 = Orthologs differentially expressed only in *C. elegans* dauers (n = 178 in *C. elegans*, Figure 1a)

(4) Class 4 = Orthologs differentially expressed only in *P. pacificus* expression profile (n = 2626 in *P. pacificus*, Figure 1a).

### Gene ontology term enrichment

To better understand which functions are enriched in these gene sets, we made use of Gene Ontology (GO) term annotations from WormBase [36]. The set of 6,126 1:1 orthologs was used as the background set, and GO annotations were assigned to *P. pacificus* genes by directly mapping them from the corresponding *C. elegans* 1:1 orthologs. Enrichment statistics were calculated with the Bioconductor package topGO [37] (see Methods). We found the biological process of “neuropeptide-signaling pathway” to be the most significantly enriched (Table 2) in the class 1 gene set, which subsumes the most conserved expression pattern across the two species. We find six out of the 16 genes belonging to this GO class to be upregulated in dauers of both *C. elegans* and *P. pacificus*, and four of them are members of “FMRF-Like Peptide” family of neuropeptides, which also have a known role in regulating pharyngeal pumping [38]. GO enrichment analysis of class 2 genes that show an opposite fold change in their expression pattern between species yields “molting cycle, collagen and cuticulin-based cuticle” as the most significantly enriched term (Table 3). This suggests that the dauers of both species possibly use different genetic components for synthesis and/or shedding of the cuticle. This potential difference in molting related cuticle processing in the two species is further supported by the observation that this GO term is also the most significantly enriched in Class 3 genes - the set of orthologous genes differentially regulated exclusively in *C. elegans* (Table 4). For class 4 genes, the most significantly enriched GO terms are mostly development related but are too broad to point to any specific function (Table 5).

**Table 2:** GO terms enriched in orthologs with same direction of fold change in both species

**GO:BP terms enriched in orthologs regulated in same direction in both species**					
**GO.ID**	**Term**	**Annotated**	**Significant**	**Expected**	**p-Value**
GO:0007218	neuropeptide signaling pathway	16	6	0.29	2.00E-07
GO:0055114	oxidation-reduction process	219	10	3.97	0.0056
GO:0006576	cellular biogenic amine metabolism	7	2	0.13	0.0064
GO:0006508	proteolysis	197	9	3.57	0.0086
GO:0046496	nicotinamide nucleotide metabolism	10	2	0.18	0.0133
GO:0009069	serine family amino acid metabolism	10	2	0.18	0.0133
GO:0019752	carboxylic acid metabolism	128	8	2.32	0.0172
GO:0009309	amine biosynthetic process	33	3	0.6	0.0212
GO:0040019	positive regulation of embryonic development	13	2	0.24	0.0222
GO:0008202	steroid metabolic process	14	2	0.25	0.0256
GO:0008033	tRNA processing	19	2	0.34	0.0455
GO:0006006	glucose metabolic process	20	2	0.36	0.0499
**GO:MF terms enriched in orthologs regulated in same direction in both species**					
**GO.ID**	**Term**	**Annotated**	**Significant**	**Expected**	**p-Value**
GO:0016616	oxidoreductase activity; acting on the CH-OH group of donors, NAD or NADP as acceptor	34	4	0.64	0.0034
GO:0016491	oxidoreductase activity	283	14	5.29	0.0107
GO:0016747	transferase activity; transferring acyl groups other than amino-acyl groups	53	4	0.99	0.0164
GO:0016831	carboxy-lyase activity	11	2	0.21	0.017
GO:0004175	endopeptidase activity	115	6	2.15	0.0193
GO:0008233	peptidase activity	180	10	3.37	0.0258
GO:0051287	NAD binding	14	2	0.26	0.0271

**Table 3:** GO terms enriched in orthologs with opposite direction of fold change in both species

**GO:BP terms enriched in orthologs regulated in opposite direction in both species**					
**GO.ID**	**Term**	**Annotated**	**Significant**	**Expected**	**p-Value**
GO:0018996	molting cycle; collagen and cuticulin-based cuticle	157	7	1.77	0.0016
GO:0010171	body morphogenesis	322	10	3.63	0.0025
GO:0040011	locomotion	789	17	8.89	0.0038
GO:0040010	positive regulation of growth rate	1024	18	11.54	0.0244
GO:0040018	positive regulation of multicellular organism growth	167	5	1.88	0.0382
GO:0019318	hexose metabolic process	28	2	0.32	0.039
**GO:MF terms enriched in orthologs regulated in opposite direction in both species**					
**GO.ID**	**Term**	**Annotated**	**Significant**	**Expected**	**p-Value**
GO:0042302	structural constituent of cuticle	21	4	0.2	3.80E-05
GO:0015078	hydrogen ion transmembrane transporter activity	30	2	0.29	0.033
GO:0050662	coenzyme binding	83	3	0.8	0.045
GO:0046912	transferase activity; transferring acylgroups, acyl groups converted into alkyl on transfer	5	1	0.05	0.047

**Table 4:** **GO terms enriched in orthologs, which are only differentially expressed in*C. elegans***

**GO:BP terms enriched in orthologs differentially expressed exclusively in C. elegans**					
**GO.ID**	**Term**	**Annotated**	**Significant**	**Expected**	**p-Value**
GO:0018996	molting cycle; collagen and cuticulin-based cuticle.	157	17	4.19	5.70E-07
GO:0040011	locomotion	789	34	21.07	0.0019
GO:0040018	positive regulation of multicellular organism growth	167	11	4.46	0.0046
GO:0006560	proline metabolic process	5	2	0.13	0.0067
GO:0010171	body morphogenesis	322	16	8.6	0.0106
GO:0009084	glutamine family amino acid biosynthesis	8	2	0.21	0.0178
GO:0006694	steroid biosynthetic process	10	2	0.27	0.0277
GO:0055114	oxidation-reduction process	219	11	5.85	0.0306
GO:0006508	proteolysis	197	10	5.26	0.0362
GO:0018991	oviposition	145	8	3.87	0.0387
**GO:MF terms enriched in orthologs differentially expressed exclusively in C. elegans**					
**GO.ID**	**Term**	**Annotated**	**Significant**	**Expected**	**p-Value**
GO:0042302	structural constituent of cuticle	21	10	0.53	1.60E-11
GO:0004222	metalloendopeptidase activity	50	6	1.25	0.0014
GO:0016776	phosphotransferase activity; phosphate group as acceptor	5	2	0.13	0.0059
GO:0016903	oxidoreductase activity; acting on the aldehyde or oxo group of donors	8	2	0.2	0.0157
GO:0003854	3-beta-hydroxy-delta5-steroid dehydrogenase activity	8	2	0.2	0.0157
GO:0019205	nucleobase; nucleoside; nucleotide kinase activity	8	2	0.2	0.0157
GO:0020037	heme binding	51	4	1.28	0.0374
GO:0004601	peroxidase activity	14	2	0.35	0.0464

**Table 5:** **GO terms enriched in orthologs, which are only differentially expressed in*P. pacificus***

**GO:BP terms enriched in orthologs differentially expressed exclusively in P. pacificus**					
**GO.ID**	**Term**	**Annotated**	**Significant**	**Expected**	**p-Value**
GO:0006898	receptor-mediated endocytosis	433	231	192.89	6.30E-05
GO:0009792	embryo development ending in birth or egg hatching	1535	740	683.81	0.00015
GO:0002119	nematode larval development	1123	549	500.27	0.00034
GO:0040020	regulation of meiosis	45	30	20.05	0.00219
GO:0042127	regulation of cell proliferation	45	30	20.05	0.00219
GO:0006412	translation	165	93	73.5	0.00691
GO:0016246	RNA interference	63	38	28.07	0.00812
GO:0055114	oxidation-reduction process	219	115	97.56	0.00912
GO:0000003	reproduction	1235	585	550.17	0.00934
GO:0040007	growth	1481	694	659.75	0.01358
GO:0009396	folic acid-containing compound biosynthetic process	5	5	2.23	0.01749
GO:0009067	aspartate family amino acid biosynthesis	10	8	4.45	0.02546
GO:0009072	aromatic amino acid family metabolic process	10	8	4.45	0.02546
GO:0006732	coenzyme metabolic process	41	27	18.26	0.0326
GO:0051603	proteolysis involved in cellularprotein catabolic process	36	24	16.04	0.03319
GO:0006396	RNA processing	72	40	32.07	0.03822
GO:0006511	ubiquitin-dependent protein catabolic process	29	18	12.92	0.04326
GO:0006418	tRNA aminoacylation for protein translation	33	20	14.7	0.04611
GO:0015684	ferrous iron transport	9	7	4.01	0.04679
GO:0042026	protein refolding	9	7	4.01	0.04679
GO:0006399	tRNA metabolic process	51	32	22.72	0.04805
**GO:MF terms enriched in orthologs differentially expressed exclusively in P. pacificus**					
**GO.ID**	**Term**	**Annotated**	**Significant**	**Expected**	**p-Value**
GO:0004298	threonine-type endopeptidase activity	13	12	5.76	4.30E-04
GO:0003899	DNA-directed RNA polymerase activity	22	17	9.74	0.00168
GO:0016491	oxidoreductase activity	283	149	125.32	0.00192
GO:0008026	ATP-dependent helicase activity	49	32	21.7	0.00229
GO:0050662	coenzyme binding	83	48	36.76	0.00828
GO:0003735	structural constituent of ribosome	93	53	41.18	0.00847
GO:0008168	methyltransferase activity	67	39	29.67	0.01438
GO:0016884	carbon-nitrogen ligase activity; with glutamine as amido-N-donor	10	8	4.43	0.02443
GO:0051082	unfolded protein binding	28	18	12.4	0.02591
GO:0030170	pyridoxal phosphate binding	34	21	15.06	0.02977
GO:0004812	aminoacyl-tRNA ligase activity	34	21	15.06	0.02977
GO:0003993	acid phosphatase activity	12	9	5.31	0.03154
GO:0031072	heat shock protein binding	23	15	10.19	0.03483
GO:0004190	aspartic-type endopeptidase activity	9	7	3.99	0.04517
GO:0015093	ferrous iron transmembrane transporter activity	9	7	3.99	0.04517
GO:0008483	transaminase activity	9	7	3.99	0.04517

### Expression cluster analysis

Apart from analyzing enrichment for GO annotations, we also performed a functional analysis based on overlap of our data with gene expression clusters in *C. elegans*, which originate from other published microarray data sets*.* To this end, we retrieved predefined expression clusters from WormBase [36] and used them for a meta-level analysis. These expression clusters represent sets of genes reported to be co-expressed under various conditions. We restricted the analysis to the set of 1:1 orthologs, and assigned expression clusters to *P. pacificus* genes by mapping annotation from the corresponding *C. elegans* orthologs. The significance scores for enrichment of each pre-defined cluster was calculated separately for the significantly up- and down-regulated genes from the dauer versus dauer exit comparison in our data (see Methods for details), and are summarized in Table 6.

**Table 6:** Expression cluster enrichment analysis

**Expression clusters over-represented in "dauer enriched" and "exit enriched genes" in C. elegans and P. pacificus**					
**Expression clusters related to dauer larvae, stress response and starvation / feeding**					
**Sl.No.**	**Expression Cluster**	**cel_dauer**	**cel_exit**	**ppa_dauer**	**ppa_exit**
1	Wang_Kim_WBPaper00005859_DauerEnriched	10.45	0	0	0
2	Wang_Kim_WBPaper00005859_EarlyGenes	0	5.99	0	10.67
3	Wang_Kim_WBPaper00005859_ClimbingGenes	0	14.58	0	4.62
4	Wang_Kim_WBPaper00005859_LateGenes	0	63.09	0	0
5	WBPaper00024393:strongly_regulated_dauer_genes_UP	1.39	0	0	0
6	WBPaper00024393:strongly_regulated_dauer_genes_DOWN	0	8.49	0	10.85
7	WBPaper00034757:up_by_oxidative_stress	4.2	0	0	0
8	WBPaper00034757:down_by_oxidative_stress	0	6.47	0	6.82
9	WBPaper00035227:heat_shock_regulated	9.74	0	0	0
10	WBPaper00035873:dpy-10_regulated	0	25.06	0	3.89
11	WBPaper00035873:dpy-9_regulated	0	2.02	0	1.57
12	WBPaper00035873:osm-11_regulated	0	2.02	0	1.57
13	WBPaper00035873:osm-7_regulated	0	2.4	0	2.07
14	WBPaper00035873:osm-8_regulated	0	42.83	0	4.45
15	WBPaper00035873:osmotically_regulated	0	0	0	4.65
16	WBPaper00032948:StarveUp2	6.17	0	2.5	0
17	WBPaper00032948:StarveUp3	7.15	0	0	0
18	WBPaper00032948:StarveUp4	10.45	0	0	0
19	WBPaper00032948:FedUp	0	78.95	0	5.12
20	WBPaper00032948:MoltOssilate	0	74.78	0	0
21	WBPaper00032062:age_regulated_genes	0	5.37	0	4.75
**Expression clusters related to to pathogen response, RNAi machinery etc.**					
**Sl.No.**	**Expression Cluster**	**cel_dauer**	**cel_exit**	**ppa_dauer**	**ppa_exit**
22	WBPaper00028482:PA14_upregulate	0	0	0	2.03
23	WBPaper00028789:PA14_vs_gacA_downregulated_4hr	0	5.9	0	0
24	WBPaper00028789:PA14_vs_gacA_downregulated_8hr	0	6.7	0	0
25	WBPaper00028789:PA14_vs_OP50_downregulated_8hr	0	6.85	0	1.81
26	WBPaper00028789:PA14_vs_OP50_upregulated_4hr	0	0	0	1.4
27	WBPaper00028789:PA14_vs_OP50_upregulated_8hr	0	0	0	2.02
28	WBPaper00030985:Enterococcus_faecalis_upregulated	0	0	0	1.6
29	WBPaper00028789:pmk-1_downregulated	0	0	0	1.46
30	WBPaper00029437:dcr-1_upregulated	3.13	0	0	0
31	WBPaper00029437:rde-4_upregulated	0	0	0	1.69
32	WBPaper00027111:eri-1(mg366)_downregulated	0	2.23	0	0
33	WBPaper00027111:rde-3(r459)_upregulated	0	0	0	1.52
34	WBPaper00035892:KIM5_regulated	0	0	0	4.79
35	WBPaper00035892:KIM5_vs_OP50_Up	0	0	0	2.36
**Tissue specific, and other expression clusters**					
**Sl.No.**	**Expression Cluster**	**cel_dauer**	**cel_exit**	**ppa_dauer**	**ppa_exit**
36	WBPaper00030839:Embryo_Pan_Neuronal	0	0	6.67	0
37	WBPaper00030839:Larval_Pan_Neuronal	4.75	0	2.71	0
38	WBPaper00031003:0hr_muscle_depleted	0	2.99	0	0
39	WBPaper00031003:24hr_muscle_depleted	0	2.11	0	2.73
40	WBPaper00031003:total_muscle_depleted	0	2.29	0	0
41	WBPaper00031003:total_muscle_enriched	0	0	2.42	0
42	WBPaper00031532:Larva_Pan_Neuronal_Depleted	0	28.03	0	11.41
43	WBPaper00031532:Larva_Pan_Neuronal_Enriched	2.48	0	4.69	0
44	WBPaper00026980:intestine_enriched	0	0	3.46	1.5
45	WBPaper00024671:AFD_AWB_vs_unsorted_downregulated	0	3.02	0	3.59
46	WBPaper00031832:slr-2_regulated	0	0	0	2.75
47	WBPaper00033101:spr-5_regulated	0	6.44	0	0
48	WBPaper00034739:N2lessDR1350	0	39.13	0	0
49	WBPaper00034739:RIL17lessRIL14	0	18.11	0	0
50	WBPaper00035905:FBF-1_Associated	0	0	5.54	0
51	WBPaper00037611:RNP-8-associated	0	0	4.92	0
52	WBPaper00025032:PAL-1_target_genes	0	2.58	0	0

This table lists - log10 transformed p-values of tests for enrichment of dauer and dauer-exit genes from both species for 52 out of 169 expression clusters. These 52 expression clusters passed the FDR corrected p-value threshold of 0.05 for at least one condition. The overlap between dauer enriched clusters and dauer-exit enriched clusters is statistically significant (Fisher’s exact test p-values = 0.0185 and 6.833E-06 respectively).

These significantly enriched clusters enable us not only to identify system-wide trends in our data but also validate them against existing data-sets. For example, we see a highly significant overlap between our *C. elegans* data and previously reported expression profiles of dauers and dauer-exit stages [5,9]. Specifically, the cluster “Dauer enriched” identified by Wang and Kim in their time-course study [5] is enriched in genes called up-regulated in our data (Table 6, row 1, column “cel_dauer”). Furthermore, other clusters from later dauer-exit time points in the same study show a significant overlap with genes called over-expressed in our dauer-exit samples (Table 6, rows 2 to 4, column “cel_exit”), thus indicating good agreement between the two data-sets. Interestingly, the clusters corresponding to “early” and “climbing” genes in *C. elegans* (Table 6, rows 2 and 3) also show a significant overlap with genes over-expressed in our *P. pacificus* dauer-exit samples (column “ppa_exit” in Table 6), pointing towards a conservation of a part of the active transcriptome during dauer recovery in the two species. A similar trend is also seen for clusters obtained from a microarray study of dauers from daf-c/TGF-beta mutants [9] (Table 6, rows 5 and 6). We also see a significant overlap between genes regulated in response to heat shock, oxidative stress, osmotic stress and response to pathogens, highlighting the fact that activation of stress response pathways is a common feature of dauer stage in both *C. elegans* and *P. pacificus*. Further, genes activated in response to starvation and on resumption of feeding are also found to be enriched in dauer and dauer-exit stages respectively for both the species, in good agreement with non-feeding status of dauers and resumption of a feeding program during dauer-exit. We observe a significant overlap between the expression clusters that are enriched in the dauers of both the species (Fisher’s exact test p-value = 0.0185), indicating a conserved signature of gene expression. This overlap of expression clusters is stronger for the dauer-exit stages of both the species (Fisher’s exact test p-value = 6.833E-06), indicating that similar transcriptional programs are activated during dauer recovery in both the species. In summary, in spite of the low overlap and correlation between differentially expressed genes in the two species, functional analysis based on GO and expression cluster enrichment could identify conserved aspects of the transcriptional program associated with the dauer recovery process in the two species.

Apart from the differentially expressed genes in the limited set of 1:1 orthologous genes, there is a comparable number of differentially expressed genes for which either no orthologs exist in the other species or the orthology cannot be uniquely resolved (2,129 *P. pacificus* specific genes versus 2626 + 184 = 2810 1:1 orthologs in *P. pacificus*, 555 *C. elegans* specific genes versus 178 +184 = 362 1:1 orthologs in *C. elegans* data, Figure 1a). This indicates that the non-conserved part of the active transcriptome show a similar dynamic like the conserved part and might play a significant role in the biology of dauer larvae of the two species. Further functional studies would be required to understand the adaptive significance of these species-specific genes.

### PFAM protein domain based analysis identifies potentially conserved and diverged functional gene classes

**Figure 2:**
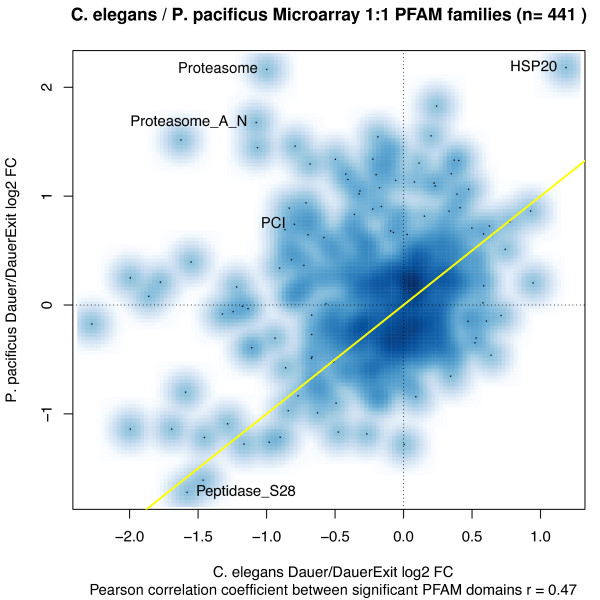
**Log2 fold changes of median PFAM gene expression levels in*P. pacificus*and*C. elegans.*** All genes on the microarrays were stratified by PFAM domain annotation into partially overlapping gene families with at least 5 members. A positive log2 fold change indicates that the corresponding genes were up-regulated in the dauer stages in comparison to the dauer exit time point.

We further investigated the functional differences in the two transcriptomes by stratifying gene expression based on their protein domain annotations. This approach has the benefit of not being dependent on orthology relationships (like some of the analyses performed above), and can identify conserved functional signatures if the same gene function is carried out by different paralogs in the two species. For this analysis, we inferred possible gene functions by annotating 13,344 gene loci in *C. elegans* and 14,018 gene loci in *P. pacificus* with PFAM protein domains (p-value of domain match < 0.001). We then stratified the gene sets from the two species into gene families by PFAM domains with at least five members on each microarray. These 441 gene families may partially overlap and are represented by their median log2 fold expression changes. The Pearson's correlation coefficient of log2 fold changes is r = 0.52 for the dauer versus dauer exit comparison (Figure 2), an improvement over the aforementioned correlation values for 1:1 orthologs (Figure 1b). Some protein domains indeed show concordant expression in both the species, such as the HSP20 family of heat shock proteins that is induced in dauers of both the species (Figure 2) and potentially confers stress resistance. Similarly, the Peptidase_S28 family of proteins is repressed in the dauer stage of both species possibly because it contains proteins with lysosomal activity, which might not be required during the repressed metabolic state of dauers [7,39]. Interestingly, we also find some protein domains with totally opposite expression patterns, such as some of the proteasome subunit domains. The genes with domains “Proteasome” and “Proteasome_A_N” are induced strongly in *P. pacificus* dauers but are down regulated in *C. elegans* dauers. Since proteasome function has been implicated in regulation of longevity [40-42], up regulation of these specific proteasome domains in *P. pacificus* might be correlated with the increased longevity of *P. pacificus* dauers versus *C. elegans* dauers [43]. Thus, in summary, PFAM annotation based analysis of the two transcriptomes also identifies some conserved functional signatures as well as some divergent signatures. This analysis also provides an important catalog of gene function, and the role of genes corresponding to the protein families identified here can be studied in more detail in *P. pacificus* dauers in the future.

### Metabolic recovery during dauer exit displays different expression patterns of key enzymes in *P. Pacificus*

**Figure 3:**
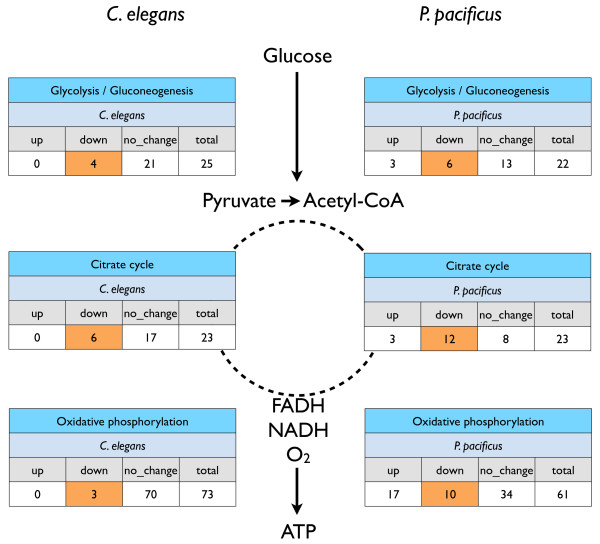
**Differences in regulation of metabolism related genes during the dauer exit time course in*C. elegans*and*P. pacificus.*** Gene-to-pathway assignments were retrieved for *C. elegans* from the KEGG database. Pathway assignments were transferred to *P. pacificus* by mapping 1:1 orthologs. All tables show genes that are up, down or at a similar expression level in a dauer vs. dauer exit (12 hours post induction) comparison.

Dauer larvae of *C. elegans* are known to undergo a remarkable shift in their intermediary metabolism to survive long periods without actively feeding [7,39]. We therefore looked in more detail into expression changes in metabolism related genes using the KEGG database [44]. Gene-pathway assignments were obtained for *C. elegans* from KEGG and transferred to *P. pacificus* via the 6,126 orthologs identified through best pairwise BLAST mapping. For each KEGG pathway, we determined the number of genes from the 1:1 orthologous set that are upregulated, downregulated or show no differences in expression levels in the dauer versus dauer-exit comparisons in both species. We observed that the three key processes of central carbon metabolism, namely glycolysis, the Krebs (TCA) cycle and oxidative phosphorylation, show entirely different dynamics during the dauer-exit time course in the two species (see Figure 3). For *C. elegans*, mRNA abundance levels for most of the genes in the three pathways are found to be similar in the dauers and dauer-exit stage (category “no_change”, Figure 3). Only a few genes are downregulated, while none of the genes is upregulated in *C.elegans*. In contrast, we see a more dynamic regulation of metabolic pathways in *P. pacificus*, with comparatively larger number of downregulated genes as well as a few upregulated genes. These differences in metabolic recovery in *P. pacificus* could have an obvious and trivial explanation in that *P. pacificus* might have a different developmental rate compared to *C. elegans*. However, as described above, several morphological and developmental traits suggest that the course of post-dauer development in *P. pacificus* and *C. elegans* is very similar*.* Hence, we hypothesize that metabolic regulation in *P. pacificus* dauers might itself be inherently different from that in *C. elegans*. This would also be consistent with the dauer recovery studies showing that *P. pacificus* dauers can survive much longer than *C. elegans* dauers [43], one of the possible reason being their ability to metabolize stored fats at a different rate and/or in a different way. Our genome-wide expression studies reflect this potential difference as divergence in pathway expression profiles between the two species. Since gene expression profiles are dynamic and can be sensitive to differences in developmental timing (e.g. [11]), more fine-grained and detailed functional studies will be needed to verify whether these differences are really due to inherent metabolic differences or an artifact of slight variations in developmental timing.

### Genes acquired by horizontal gene transfer and orphan genes in *P. Pacificus* are developmentally regulated

Previous studies have identified genes in *P. pacificus* that likely originate from lateral gene transfer events. For example, *P. pacificus* is the first nematode, which is not a plant parasitic species yet contains cellulase genes [25]. *P. pacificus* cellulase genes are most similar to genes found in the social amoeba of the Dictyostelid group [45]. These cellulases are fully functional and have been maintained over long evolutionary time periods [46]. We have identified seven cellulase genes in the current transcriptome annotation, six of which are represented on our *P. pacificus* microarrays. In a dauer versus mixed-stage comparison, four of these cellulases are significantly differentially expressed, all of them being down regulated in dauers (or equivalently, expressed predominantly in non-dauer mix-stages, Additional file 4: Table S3). This is in agreement with their hypothesized function of enabling feeding on novel food sources [46]. For example, one gene (Contig66-snapTAU.4) is already significantly up-regulated as early as 12 hours post induction (Additional file 4: Table S3, column logFC(D0/D12)).

Another interesting gene family, which was most likely acquired from beetles, is the diapausins. These genes encode small peptides, which are thought to protect the dormant beetle against microbial infections in diapause. Diapausins provide antifungal activity by acting as Ca2+ channel blockers [47]. Out of five members of this gene family in *P. pacificus* genome, three are represented on our microarrays. Two of them are significantly up-regulated in dauers compared to dauer exit (Additional file 4: Table S4, column logFC(D0/D12)) and might be important for antifungal immunity in the dauer stage on the beetle, while the third gene does not respond to the dauer exit program. This gene is in fact downregulated in a dauer versus mix-stage comparison (column logFC(D0/MixStage), Additional file 4: Table S4) suggesting potential anti-fungal role in other developmental stages. Thus, while previous studies in our lab have demonstrated that both gene families (cellulases and diapausins) originate from lateral gene transfer events, we are now able to show members of both gene families are developmentally regulated in agreement with their proposed functions: cellulase genes are implicated in feeding and are hence downregulated in dauers, and at least two diapausin gene appears to have a role in protecting hibernating stages. These hypotheses generated from our microarray data can further be investigated at a functional level.

One third of all *P. pacificus* genes are pioneer genes, which do not show any obvious sequence similarity to other organisms [26]. On average, pioneer genes are significantly higher expressed in the dauer stage in comparison to the remaining genes (p-value < 1.0E-06; Additional file 1: Figure S3). We speculate that pioneer genes could be especially important to the evolution of the dauer stage in *P. pacificus,* which is essential for its necromenic life style.

## Discussion

The dauer is an ecologically regulated developmental stage observed in many free-living nematode species and hence provides an interesting model to investigate how ecological adaptations are integrated into developmental pathways during evolution [1]. Extensive studies in *C. elegans* have uncovered the genetic regulators of dauer formation and comparative studies from other free living nematodes have begun to provide insights into evolution of dauer regulatory genes and pathways [33,34,48]. *P. pacificus* presents an ideal model for comparing dauer larvae of free living nematodes. In this study we provide a comprehensive comparison of gene expression in the dauer stage and dauer exit (12 hour post induction) of the two nematode model systems *P. pacificus* and *C. elegans* using the Agilent microarray platform. While we are aware of the available *C. elegans* expression data on dauer development (e.g. [5,6,8-14], we felt that it was necessary to generate *C. elegans* data *de novo* for two reasons. First, we wanted to use the same platform as that used for the analysis of *P. pacificus* in order to enhance the power of a direct comparison. Second, we wanted to benefit from technical advances in custom and long nucleotide microarrays. Based on cross-species comparison of transcriptomes of the ecologically important dauer stage, we draw four major conclusions from our studies.

First, we provide a list of similarities and differences between *P. pacificus* and *C. elegans*, which shows an unexpected level of divergence at transcriptome level, even though the dauer stage and recovery process appear to be developmentally conserved. While this comparison allows several evolutionary conclusions (see below), the *P. pacificus* data set on its own can be used as a starting point for a functional analysis of the dauer stage and dauer exit. This data set represents an invaluable resource given the importance of the dauer stage for the ecology of this nematode. The association of *P. pacificus* with scarab beetles is restricted to the dauer stage as long as the beetle is alive [27,28]. Only after the beetle´s death, the nematode exits from the dauer stage to feed on the microbes, which develop on the carcass of the insect. Thus, the *P. pacificus* dauer stage has a well-defined ecological niche and the expression profiles described in this study will serve as an entry point to future functional studies. For example, our transcriptomic data identifies many *P. pacificus* specific genes as upregulated in the dauer stage, which implies a potential function in adaptations enabling survival on beetles.

Second, we show that metabolic differences exist between both species, with different patterns of regulation of genes involved in the central carbon metabolism pathways (glycolysis, tricarboxylic acid cycle and oxidative phosphorylation) of *P. pacificus* upon dauer exit. This difference does not result in obvious phenotypic changes during dauer exit, but may be linked to the differences in life history traits. This is especially relevant given that *P. pacificus* is adapted for longevity [43]. Under experimental conditions, *P. pacificus* survives for up to one year in the dauer stage, whereas *C. elegans* N2 dauer larvae die after approximately 22 weeks. Further studies will reveal how much of the observed metabolic differences are explained by different life-history traits versus differences in rate of development.

Third, this study provides the first nematode evo-devo comparison looking at the downstream consequences of homologous developmental processes between species belonging to different nematode genera. While detailed studies between nematodes as distinct as *C. elegans* and *P. pacificus* have investigated the regulation of vulva and gonad development, sex determination and dauer formation [30,34,49,50], most of these studies are concerned with the regulatory mechanisms rather than the “executional programs” of the corresponding developmental processes. The divergence in the expression profiles of *C. elegans* and *P. pacificus* adds an important new finding to the growing literature of evo-devo. Previous studies have indicated the limited conservation in the genetic and molecular control of developmental processes in *P. pacificus* and *C. elegans*. For example, vulva induction relies on different signaling pathways, requires a novel regulatory linkage and the acquisition of novel protein domains in *P. pacificus* Wnt signaling [32]. This type of result has been discussed as an example for the theory of developmental systems drift, which proposes that conserved developmental and morphological structures can be regulated by largely diverse regulatory mechanisms [51]. Considering that gene regulatory networks are hierarchically structured, with possibly different rates of evolution at the top level regulatory genes and the most downstream level of effector genes [52], it can be argued that in principle, developmental systems may diverge due to differences/drift at any of these levels. Unfortunately, unlike the top-level regulatory network, the downstream effector programs of vulva development have largely escaped identification by developmental genetic approaches and have not been easily accessible to transcriptome studies as they are single-cell or small group of cell responses. Our study circumvents this limitation because dauer formation is a “whole body response” of the organism to harsh environmental conditions.

The dauer context is also interesting because in *P. pacificus*, the key transcription factors of the dauer regulatory network, DAF-16 and DAF-12, are conserved [33,34]. However, this in itself does not indicate the extent to which the downstream targets of the regulatory network are subject to evolutionary change. Herein, we could demonstrate for the first time that the core downstream execution program of a developmental stage can differ tremendously between *P. pacificus* and *C. elegans,* in spite of conservation of upstream regulators like DAF-16 and DAF-12. Thus, these observations make a case for extending the concept of developmental systems drift to the downstream molecular execution of specific developmental stages.

The fourth conclusion is also related to evolutionary theory. While future studies will have to reveal how much of the observed differences between *P. pacificus* and *C. elegans* is really of functional importance, it has often been assumed that such differences might simply be neutral [23,53]. Gene expression, which is neither strongly deleterious nor advantageous, previously termed “gratuitous expression" [53], will not be under selection and will be free to evolve by drift. Consequently, such expression is probably not functional. However, such arguments may not apply to the dauer stage since nematodes live off their internal limited energy resources and any random or neutral transcriptional activity would diminish these limited resources.

Comparative studies in developmental genetics have driven the studies on evolution of developmental mechanisms, and with whole genome sequencing of many animal species has now highlighted new facets of evolutionary dynamics through comparative genomic studies [21]. Since transcriptional regulation is a key building block in the genotype to phenotype translation, comparative transcriptomic studies add another dimension to the analysis of evolutionary processes [23]. Our work contributes to the growing set of results from comparative transcriptomics in diverse developmental systems (e.g. [54-58]). These studies together span a range of conclusions, from high transcriptomic conservation at one end, to relatively low conservation in others, suggesting inherent constraints as well as flexibility in the evolution of gene regulatory networks [23]. Future studies comparing transcriptomes of homologous biological processes in related species, will be important for understanding the role of transcriptome evolution in generating animal diversity. Ultimately, this will also reveal the extent to which the conserved or divergent expression changes are subject to adaptive and non-adaptive forces during evolution.

## Methods

### Worm strains and culture

We used wild-type strains of two distinct nematode species in all of our experiments. For *Caenorhabditis elegans*, we used the N2 (Bristol) strain. For *Pristionchus pacificus*, we used the RS2333 strain (formerly known as PS312). For mixed stage cultures, 10 to 15 early adults were spotted on 10 cm NGM plates and allowed to grow at 20°C for 5 days, and washed off with M9 for RNA extractions. The dauers for both species were obtained from liquid cultures grown at 25°C. For this, 15 to 20 mixed stage plates (see above) were washed off and suspended in a final volume of 500 ml of S-medium in a 3000 ml Erlenmayer flask. 200ul Nystatin (50 mg/ml stock in DMSO) and 500ul Kanamycin (20 mg/ml stock solution) was added to prevent fungal and bacterial contamination. On day 1, 5 and 8 of culture, 12.5 ml of OP50 (20% w/v stock in S-medium) was added as food source. The cultures were grown for 12 to 14 days in a shaker incubator @ 220 rpm, 25°C. Dauers were purified from liquid cultures between days 12 to 14. The culture was centrifuged to obtain a worm pellet, which was then incubated with 1% SDS for 30 minutes to kill any non-dauers. Live worms were separated by sucrose flotation, and a subsequent precipitation in 15% Ficoll400 resulted in a pure dauer pellet (confirmed under a stereo microscope). The washed dauers were incubated overnight in 0.1 M NaCl at 20°C, to let them recover from any possible stress induced during the harsh dauer purification process. The dauers were then collected by centrifugation, and used for RNA extraction and for starting dauer-exit cultures.

### Dauer exit time course

For the dauer-exit 12-hour time-point samples, around 200 purified dauers were spotted on each 10 cm NGM plate pre-spotted with 2 ml OP50, and grown at 20°C for 12 hours. These conditions ensured abundant food supply and non-crowded conditions, favorable for inducing dauer exit [3]. Worms from 20 to 30 such plates were used for RNA extraction per sample. The 12-hour time-point was chosen for both the species to give them sufficient time to recover from dauer stage and manifest measurable transcriptional changes. The recovering worms from both species were monitored to ensure comparable developmental rates based on the following criteria: (1) Most of the population (~90%) of the recovering worms from *C. elegans* as well as *P. pacificus* resume pharyngeal pumping within 3 hours after inducing dauer exit. (2) At the 12 hour time-point (the stage of dauer-exit samples), no discernible morphological differences are found between the recovered dauers from both species. (3) The recovering animals from both the species enter a lethargus between 13 to 14 hours post-recovery [3]. (4) The worms that are allowed to develop further at 20^0^ C reach the next moult (L4 for *C. elegans,* J4 for *P. pacificus*) between 22 to 23 hours after induction of dauer exit. (5) Finally, recovered worms from both species started laying eggs between 42 to 45 hours post-induction.

### RNA extraction

Total RNA was isolated using TRIzol (Invitrogen) according to the manufacturer's instructions. Four biological replicates for each stage (mixed, dauers, dauer-exit at 12 hrs) were prepared from parallel cultures. The extracted RNA was purified by using phenol:chloroform:isoamyl alcohol precipitation, to remove any trace of TRIzol contamination which might interfere with subsequent reactions. The RNA pellet was suspended in RNAse free water, quantity and quality was assayed on a Nanodrop spectrophotometer, and stored at −80°C until the microarray reactions.

### Microarray design and experiments

We used the Agilent *C. elegans* oligonucleotide microarrays (~ 43,000 probes for ~ 20,000 open reading frames, GEO accession: GPL10094) for all *C. elegans* experiments. For *P. pacificus*, we designed custom Agilent microarrays based on the most recent transcriptome annotation (~ 23,000 gene predictions, predicted with external evidence from 454 EST alignments). We could accommodate ~ 93,000 probe sequences on our custom *P. pacificus* microarrays (NCBI GEO platform accession GPL14372). This probe set was designed using the OligoWiz 2.0 software [59] and submitted for custom fabrication to Agilent Technologies via their eArray web tool. Out of 93,000 probes, 87,070 probes map uniquely to the latest genome assembly [26]. We restrict all subsequent analyses to a probe set of 69,916 high-confidence probes.

Microarray hybridizations were carried out in a two-color format, with 4 biological replicates per experiment including a pair of dye-flips. Two sets of hybridizations were carried out: (1) Dauers versus Mix-Stage, and (2) Dauer-Exit at 12 hr versus Mix-stage, thus making a total of 8 microarray hybridizations per species. Equal amounts of total RNA (500 ng to 1000 ng) for 4 biological replicates from each stage was used to prepare Cy5- or Cy3- labelled cRNA using Quick Amp Labelling Kit (Agilent Technologies, Inc, Santa Clara, CA, USA), as per manufacturer's instructions. Based on the starting amounts of total RNA, appropriate dilutions of positive controls (Spike Mix-A and Spike-Mix B from Agilent) had been added to the reaction-mix before the RT reaction, as per manufacturer instructions. The labelled cRNAs were hybridized either to Agilent *C. elegans* oligonucleotide microarrays or our custom *P. pacificus* microarrays from Agilent. The arrays were scanned using GenePix 4000B Microarray Scanner, and raw data extracted using GenePix Pro sofware (version 6).

### Analysis of microarray expression data

The data was analyzed using the Bioconductor [60] package limma [61]. Briefly, the array quality was assessed by checking for uniform background and foreground intensities over the entire array. The signal was background corrected using the normexp method [62]. The arrays were lowess normalized individually (“normalizeWithinArrays” option), with differential weights assigned to probes and to positive control spike-ins, which are expected to show no fold change [63]. This differential weighing of probes is particularly necessary to account for changes in the relative proportion of mRNA versus total RNA. Without this differential weighing scheme, the fold change calculations can be erroneous [64]. The weight parameters were optimized based on MA-plots such that spike-in controls show their expected fold change values. lmFit function was used to fit a linear model to probe intensities across arrays, and differential expression was calculated by empirical Bayes method using the eBayes function [65]. Control of FDR was employed as correction for multiple testing. All the data from this publication have been deposited in a MIAME compliant format [66] at NCBIs Gene Expression Omnibus database (http://www.ncbi.nlm.nih.gov/geo/) and are accessible through GEO Series accession numbers GSE30977 and GSE31861.

### Mapping of 1:1 orthology relations

We used a pairwise best BLASTP strategy to compute 1:1 orthologs. Briefly, we ran all protein sequences from *C. elegans* as query versus the database of *P. pacificus* gene predictions and vice versa. Only hits with a BLAST score > = 50 bits were retained. We define mutually best hits as 1:1 orthologs. We identified 7,176 ortholog pairs with this methodology.

### Gene ontology enrichment analysis

GO ontologies for *C. elegans* were downloaded from wormmart WS200. Gene ontologies were assigned to *P. pacificus* genes based on the previously defined 1:1 orthologs. The topGO tool [37] (version 2.4.0) was used for computing significantly enriched GO terms. We used the “GOFisher” test statistic and a p-value cutoff of 0.05. The background set was limited to the 6,126 orthologs that are represented on both *C. elegans* and *P. pacificus* microarrays. We did not apply any multiple testing correction to the reported p-values. We followed this strategy to uncover all “trends” in the data to attain a comprehensive picture of the underlying biology.

### Expression cluster enrichment analysis

WormBase [36] contains information on co-expressed gene groups in *C. elegans*. The list of microarray experiments where a given *C. elegans* gene is known to be differentially expressed can be extracted from the section “Expression Cluster” from the WormBase gene summary page for each gene. We retrieved all available expression clusters for *C. elegans* genes from the WormBase web site. We inferred expression clusters for *P. pacificus* based on the set of 1:1 orthologs. Only the clusters associated with the 6,126 1:1 orthologs were used as the background set. P-values for expression cluster enrichment in dauer-enriched or dauer exit-enriched gene sets were computed with a 2x2 Fisher exact test, and a multiple testing correction to control FDR was applied.

### Pfam domain annotation

We annotated the proteome of *C. elegans* and *P. pacificus* with PFAM domain matches (PFAM release V23/4 [67])). Protein HMM searches were performed with HMMer 3.0 [68] using a p-value cutoff of 0.001.

### KEGG pathway analysis

We retrieved the latest gene to pathway mapping for *C. elegans* from the KEGG SOAP server (see http://www.genome.jp/kegg/soap for details). Again, pathway annotations were transferred to *P. pacificus* with the help of the 1:1 orthologs. Gene expression information and pathway information were overlaid to generate Figure 3.

### Data access

Microarray data have been deposited in NCBIs Gene Expression Omnibus database (http://www.ncbi.nlm.nih.gov/geo/) and are accessible through GEO Series accession numbers GSE30977 and GSE31861. The gene set annotation for this manuscript may be obtained from http://www.pristionchus.org/download/ . The list of 1:1 orthologs and PFAM domain annotations may be also obtained from there.

## Competing interests

The authors declare that they have no competing interests.

## Authors’ contributions

AS, RJS and CD designed the experiments and wrote the manuscript. AS performed the experiments. AS and CD analyzed the data. All authors read and approved the final manuscript.

## Supplementary Material

Additional file 1 **Figure S1.The impact of different normalization strategies on expression fold change estimation. a) The general concept of using spike-ins to control for changes in total mRNA abundance relative to total RNA.** See van de Peppel *et al.*, 2003 for a detailed explanation. b) MA-plot of a Loess based normalization assuming a net mRNA expression fold change of 0 between the two samples. The M axis depicts the log2 fold change between the red and green microarray channels. The A axis is the average log2 intensity across both microarray channels. The position of the spike-in signals (colored dots) clearly indicate a deviation from this assumption. The corresponding legend in the upper left shows the expected fold change of the spike-ins relative to total RNA levels. Consequently, the location of the spike-ins corresponds to a down-shift of mRNA abundance levels within the total RNA population. c) Differentially expressed gene counts for the dauer vs. mixed stage comparison conditional on the normalization strategy. Figure S2. Explanation of a common reference design for microarrays (see Eisen and Brown 1999). For each species, RNA from biological replicates of mix-stage worms (M1 to M4) were combined together to generate one common reference pool. Labelled aRNA produced from independent biological replicates were then co-hybridized with labelled aRNA from the common reference pool, including two dye-swaps. Samples D1 to D4 represent Dauer samples and DE1 to DE4 represent dauer exit samples at 12 hour timepoint. The blue arrows indicate the direction of labelling in each co-hybridization (arrow head = Cy3 labelled, arrow tail = Cy5 labelled). This design allows comparison via the common reference pool, but at the same time remains flexible for adding more time-points to the study, if needed. Figure S3. Cumulative plot of log2 expression fold changes for genes with and without sequence conservation (pioneer genes). The two distributions are significantly different (two-sample Kolmogorov-Smirnov test; p-value < 10E- 16). Click here for file

Additional file 2 **Table S1. Differential expressed genes in the dauer versus dauer-exit comparison in*P. pacificus*.** Click here for file

Additional file 3 **Table S2. Differential expressed genes in the dauer versus dauer-exit comparison in*C. elegans*.** Click here for file

Additional file 4 **Table S3. Differential expression of*P. pacificus*cellulase genes that were acquired by horizontal gene transfer.** Table S4. Differential expression of *P. pacificus* diapausin genes that were acquired by horizontal gene transfer. Click here for file
