# Supplementary material for: Divergent gene expression in the conserved dauer stage of the nematodes Pristionchus pacificus and Caenorhabditis elegans
Source: BMC Genomics. 2012 Jun 19;13:254. doi: 10.1186/1471-2164-13-254 (PMC3443458; doi:10.1186/1471-2164-13-254)
Supplement: Additional file 1 — Figure S1.The impact of different normalization strategies on expression fold change estimation. a) The general concept of using spike-ins to control for changes in total mRNA abundance relative to total RNA. See van de Peppel et al., 2003 for a detailed explanation. b) MA-plot of a Loess based normalization assuming a net mRNA expression fold change of 0 between the two samples. The M axis depicts the log2 fold change between the red and green microarray channels. The A axis is the average log2 intensity across both microarray channels. The position of the spike-in signals (colored dots) clearly indicate a deviation from this assumption. The corresponding legend in the upper left shows the expected fold change of the spike-ins relative to total RNA levels. Consequently, the location of the spike-ins corresponds to a down-shift of mRNA abundance levels within the total RNA population. c) Differentially expressed gene counts for the dauer vs. mixed stage comparison conditional on the normalization strategy. Figure S2. Explanation of a common reference design for microarrays (see Eisen and Brown 1999). For each species, RNA from biological replicates of mix-stage worms (M1 to M4) were combined together to generate one common reference pool. Labelled aRNA produced from independent biological replicates were then co-hybridized with labelled aRNA from the common reference pool, including two dye-swaps. Samples D1 to D4 represent Dauer samples and DE1 to DE4 represent dauer exit samples at 12 hour timepoint. The blue arrows indicate the direction of labelling in each co-hybridization (arrow head = Cy3 labelled, arrow tail = Cy5 labelled). This design allows comparison via the common reference pool, but at the same time remains flexible for adding more time-points to the study, if needed. Figure S3. Cumulative plot of log2 expression fold changes for genes with and without sequence conservation (pioneer genes). The two distributions are significantly different (two-sample [file 1471-2164-13-254-S1.pdf]

a)

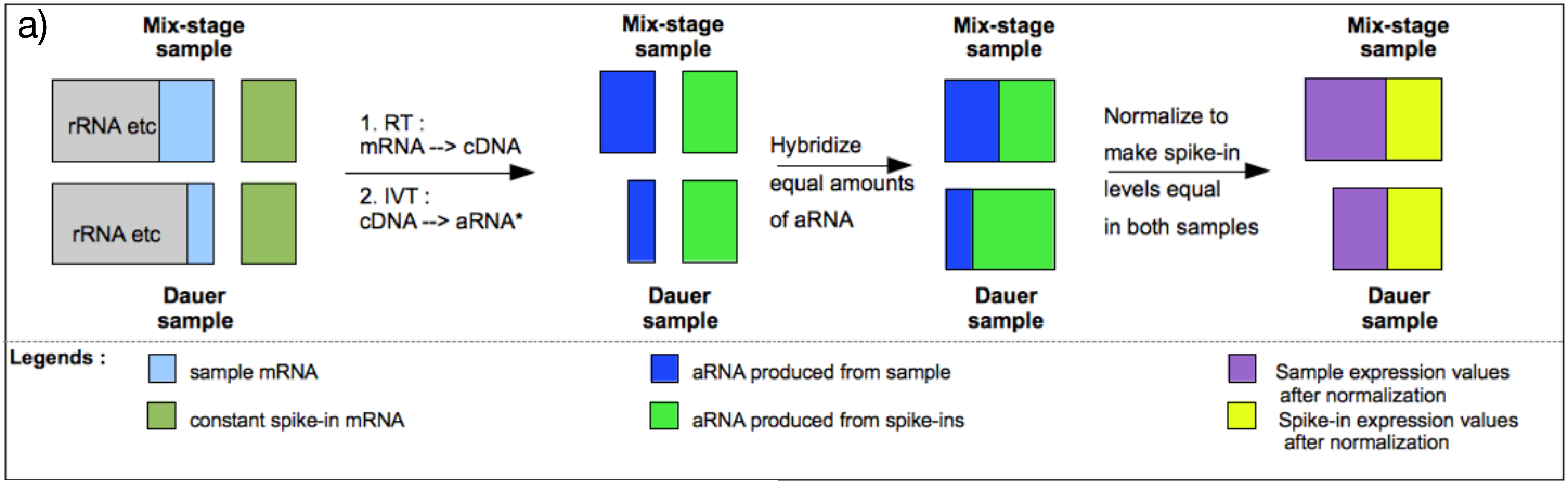

b)

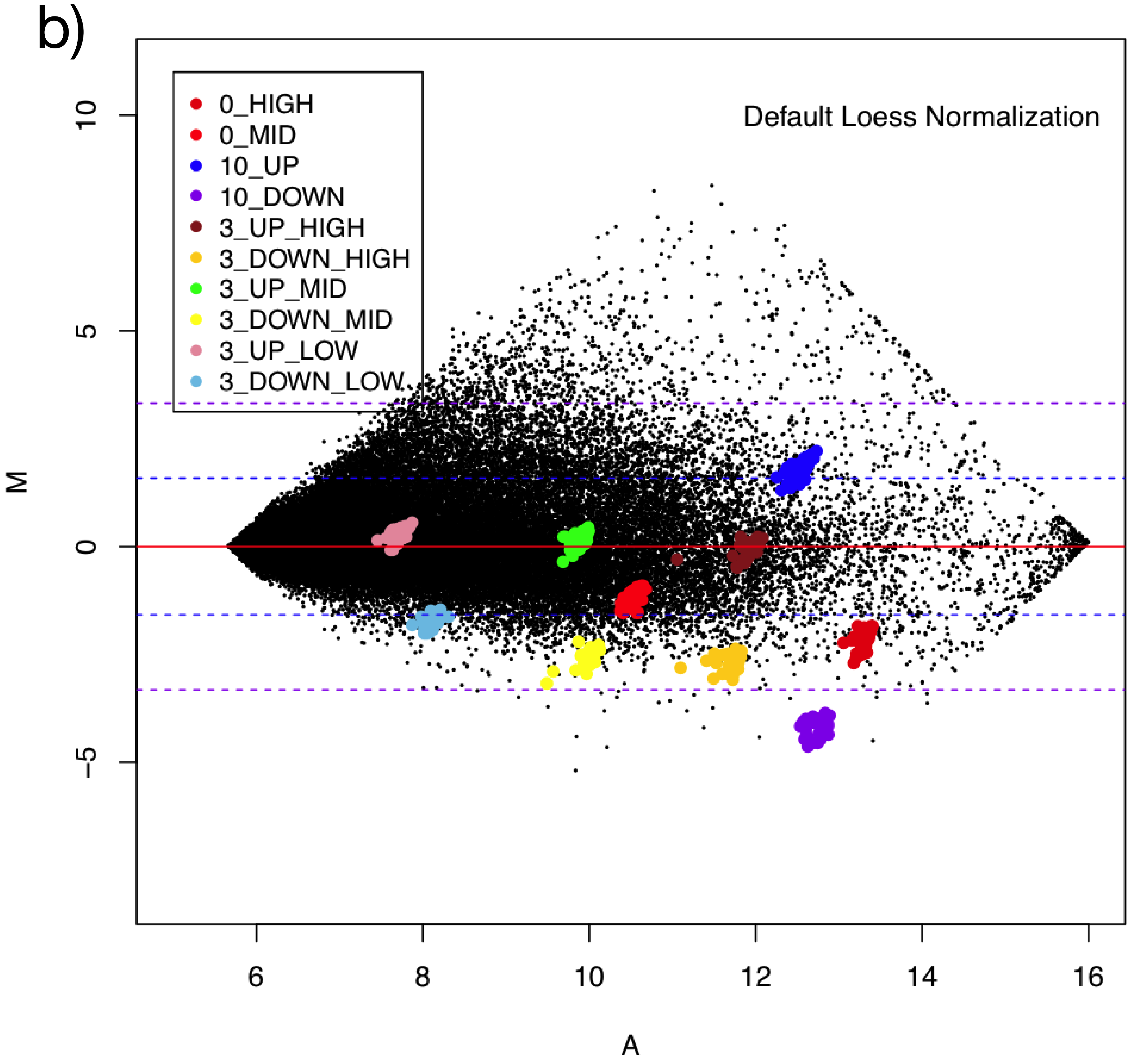

c)

C. elegans **Dauer vs Mix stage comparison**		
	Default Loess	Weighted loess
UP	1991	1099
DOWN	2681	3162
**Total**	**4672**	**4261**

P. pacificus **Dauer vs Mix stage comparison**		
	Default Loess	Weighted loess
UP	5166	3910
DOWN	5748	6121
**Total**	**10914**	**10031**

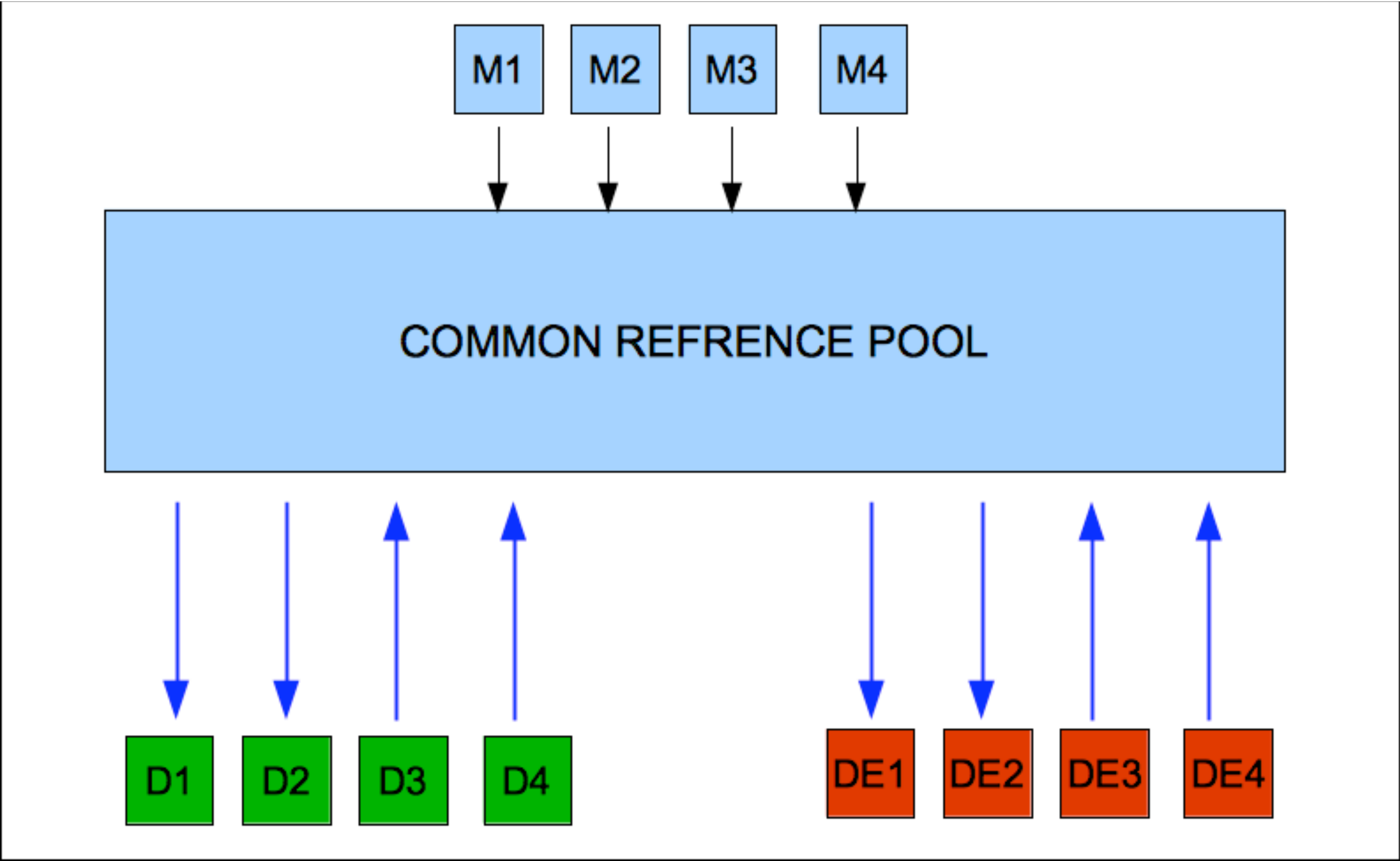
M1
M2
M3
M4
COMMON REFRENCE POOL
D1
D2
D3
D4
DE1
DE2
DE3
DE4

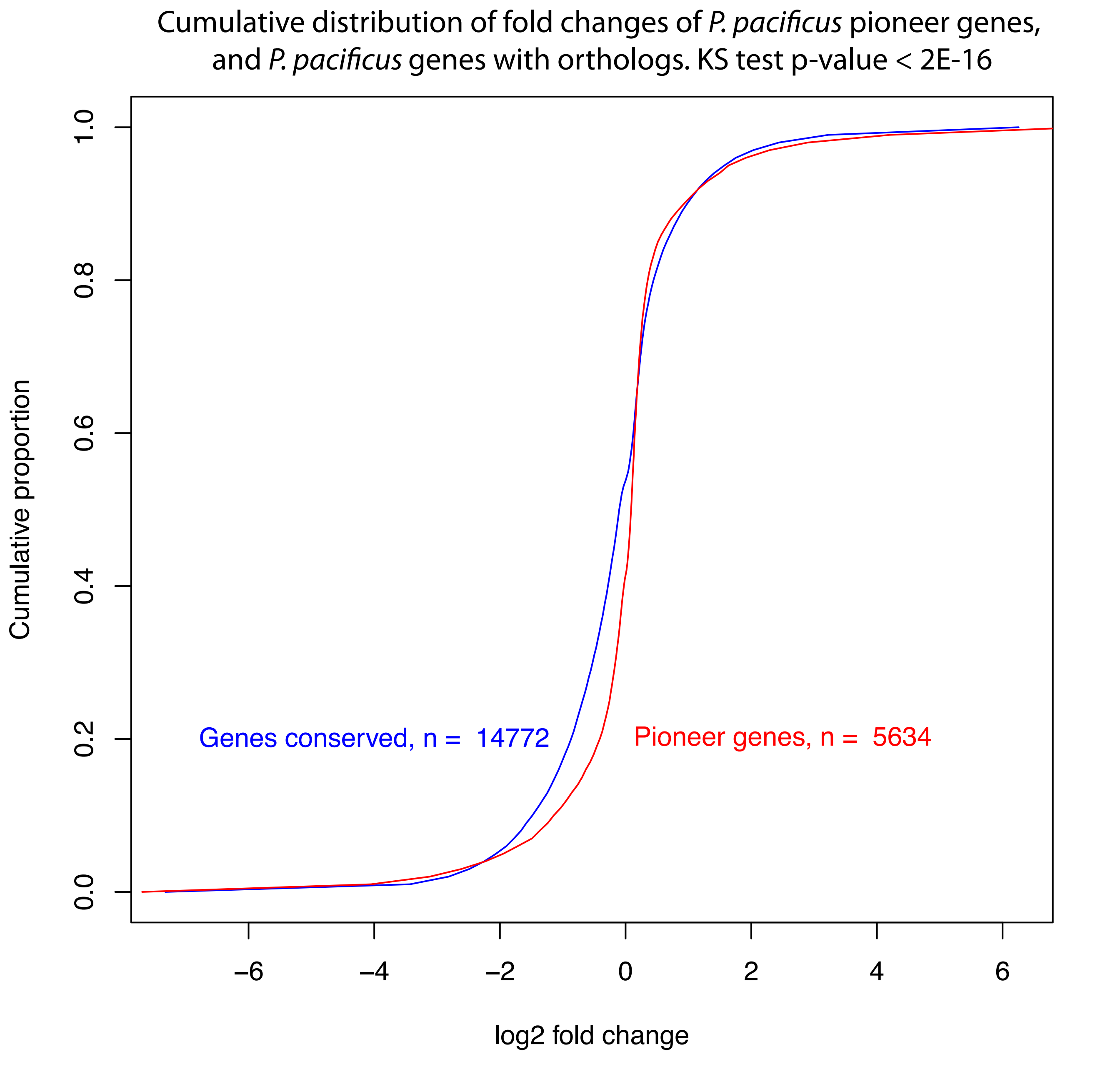
Cumulative distribution of fold changes of *P. pacificus* pioneer genes,
and *P. pacificus* genes with orthologs. KS test p-value < 2E-16
Cumulative proportion
0.0
0.2
0.4
0.6
0.8
1.0
Genes conserved, n = 14772
Pioneer genes, n = 5634
−6
−4
−2
0
2
4
6
log2 fold change
