# Supplementary material for: Divergent gene expression in the conserved dauer stage of the nematodes Pristionchus pacificus and Caenorhabditis elegans
Source: BMC Genomics. 2012 Jun 19;13:254. doi: 10.1186/1471-2164-13-254 (PMC3443458; doi:10.1186/1471-2164-13-254)
Supplement: Additional file 4 — Table S3. Differential expression ofP. pacificuscellulase genes that were acquired by horizontal gene transfer. Table S4. Differential expression of P. pacificus diapausin genes that were acquired by horizontal gene transfer. [file 1471-2164-13-254-S4.pdf]

Supplementary Table 3 : Differential expression of cellulase genes

Sl.No.	Cellulase	logFC(D0/D12)	pValue(D0/D12)	logFC(D/(MixStage)	pValue(D0/MixStage)
1	Contig66-snapTAU.4	-1.3482722	0.0008984778	-1.40083215	2.05E-05
2	Contig96-snapTAU.24	-0.358561	0.1742552558	-1.52928599	1.14E-05
3	Contig41-snapTAU.19	0.4096106	0.3367378046	-2.1315587	2.23E-05
4	Contig66-snapTAU.22	0.5897307	0.3573041471	0.5476765	1.22E-01
5	Contig4-snapTAU.543	-0.6291976	0.3954865018	-0.49346172	1.10E-02
6	Contig8-snapTAU.181	-0.0627098	0.8825763137	-0.04414294	8.48E-01

Supplementary Table 4 : Differential expression of diapausin genes

Sl.No.	Diapausins	logFC(D0/D12)	pValue(D0/D12)	logFC(D0(MixStage)	pValue(D0/MixStage)
1	Contig43-snapTAU.158	3.24066256	0.0001598057	2.152147	2.97E-05
2	Contig52-snapTAU.23	1.11845923	0.0178361106	-2.397716	1.50E-05
3	Contig0-snapTAU.430	-0.08021376	0.8471945265	-1.131421	2.25E-04
